# Acute Esophageal Necrosis Associated with *Strongyloides stercoralis* Hyperinfection

**DOI:** 10.4269/ajtmh.18-0664

**Published:** 2019-05

**Authors:** Maiko Tomori, Mitsuru Mukaigawara, Masashi Narita

**Affiliations:** 1 Department of Medicine, Okinawa Chubu Hospital, Uruma, Okinawa, Japan;; 2 Department of Anesthesiology, University of the Ryukyus, Nishihara, Okinawa, Japan;; 3 Department of Medicine, Okinawa Miyako Hospital, Miyakojima, Okinawa, Japan

**Figure 1.**
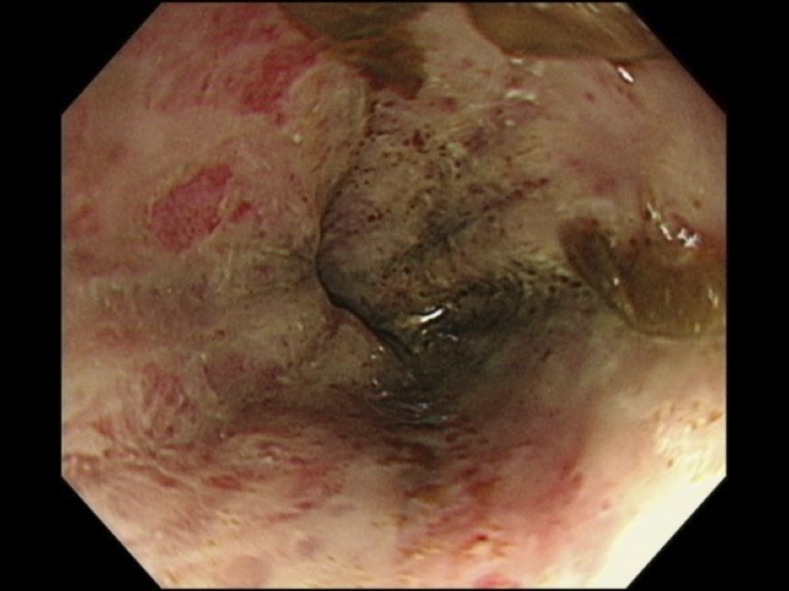
Upper endoscopy identified esophageal necrosis in the lower esophageal tract. This figure appears in color at www.ajtmh.org.

An 84-year-old woman presented to the emergency department with a 3-week history of hoarseness and decreased appetite. Four weeks before admission, she had started to take prednisolone (30 mg/day) for her unilateral facial palsy. On arrival, she was obtunded and her systolic blood pressure was 80 mmHg. Bowel sounds were reduced, and abdominal X-ray revealed gastric and small intestinal expansion. No evidence of intestinal obstruction was obtained. The upper endoscopy revealed necrotic esophagitis in the middle and lower parts of the esophagus and duodenal erosions (Figure 1).

**Figure 2.**
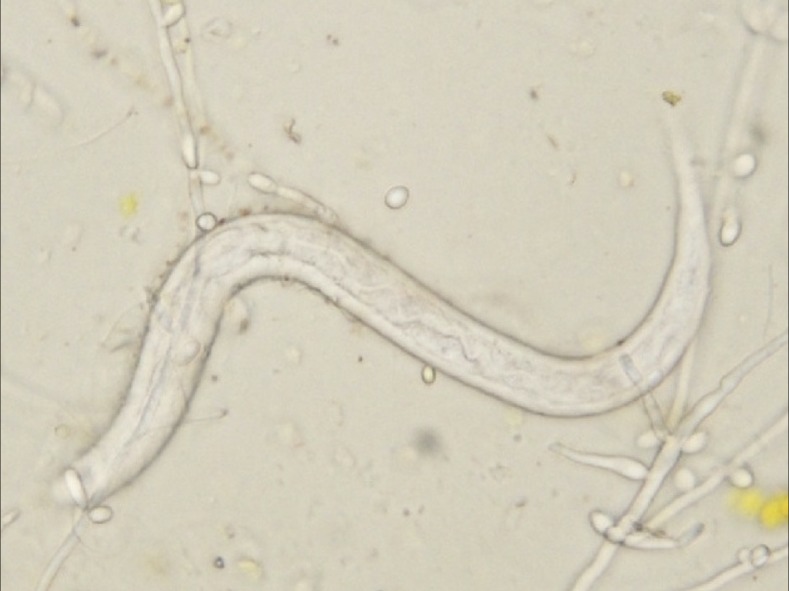
Direct microscopic examination of the duodenal fluid identified mobile larvae of *Strongyloides stercoralis.* This figure appears in color at www.ajtmh.org.

**Figure 3.**
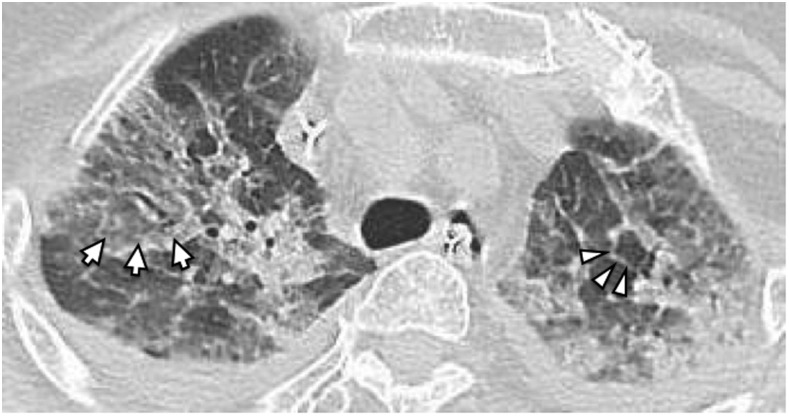
Computed tomography revealed diffuse ground-glass opacity (arrow) with interlobular septal thickening (arrowhead). It showed no findings suggestive of transmural esophageal necrosis.

Direct microscopic examination of the duodenal fluid identified many mobile larvae of *Strongyloides stercoralis* (Figure 2). Microscopic examinations of sputum samples identified immobile larvae, which suggested *S. stercoralis* hyperinfection syndrome. A computed tomography of the lungs showed multifocal ground-glass opacity primarily in the upper lobes with slight interlobular septal thickening, which is called crazy-paving appearance (Figure 3).^1^ There were no findings suggestive of esophageal necrosis. Cerebrospinal fluid analysis showed the leukocyte count of 1 cell/mm^3^ and glucose count of 133 mg/dL (blood glucose count of 265 mg/dL). Ivermectin was started via nasogastric feeding tube. Her condition did not improve with a conventional dose of ivermectin (200 mcg/kg/day for 2 days, each dose 2 weeks apart) and broad-spectrum antibiotics.

**Figure 4.**
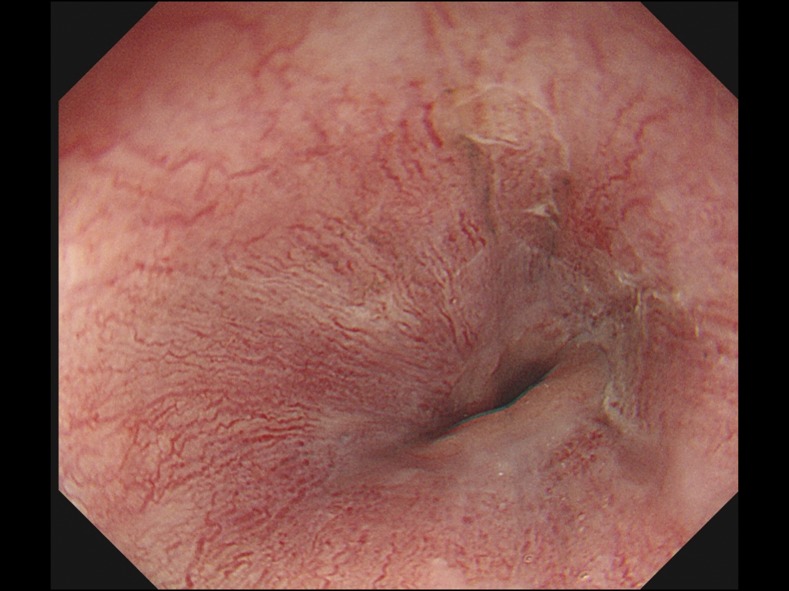
The follow-up upper endoscopy revealed improved all circumference erosion and a good epithelization of the esophagus. This figure appears in color at www.ajtmh.org.

Repetitive microscopic examinations of the duodenal fluid identified less mobile but still many larvae of *S. stercoralis*. Steroids were withheld immediately, and she was started on ivermectin for seven consecutive days, for which she responded well. A follow-up upper endoscopy revealed good epithelization of the esophagus (Figure 4). Microscopic examination of the duodenal fluid showed no larvae. Culture results of cerebrospinal fluid, sputum, urine, and blood samples were all negative. Real-time polymerase chain reaction did not detect *S. stercoralis* from urine and cerebrospinal fluid.

Strongyloidiasis is a soil-transmitted nematode infection. The distinct feature of *S. stercoralis* is autoinfection, an ability to replicate in the host without repetitive infections.^2^ When the host’s immune system is impaired, filariform larvae penetrate the intestinal wall to enter the bloodstream, causing *S. stercoralis* hyperinfection and dissemination.^3^ For this patient, recent steroid use had triggered hyperinfection.

Esophageal necrosis is caused by conditions such as multi-organ dysfunction, vasculopathy, sepsis, diabetic ketoacidosis, and malignancy.^4^ Our literature review identified no previously reported cases of esophageal necrosis associated with strongyloidiasis. In immunocompromised patients from areas of strongyloidiasis endemicity, we need to consider *S. stercoralis* hyperinfection syndrome and dissemination as an etiology of unexplained severe intestinal damage such as esophageal necrosis.
